# The optimisation of public health emergency governance: a simulation study based on COVID-19 pandemic control policy

**DOI:** 10.1186/s12992-023-00996-9

**Published:** 2023-12-04

**Authors:** Keng Yang, Hanying Qi

**Affiliations:** 1 Institute of Economics, Tsinghua University, Beijing, 100084 China; 2 One Belt-One Road Strategy Institute, Tsinghua University, Beijing, 100084 China; 3 The New Type Key Think Tank of Zhejiang Province’s “Research Institute of Regulation and Public Policy”, Zhejiang University of Finance and Economics, Hangzhou, 310018 China; 4 China Institute of Regulation Research, Zhejiang University of Finance and Economics, Hangzhou, 310018 China

**Keywords:** Public health emergencies, Prevention and control policies, Optimisation, Cost-benefit, Value of health

## Abstract

**Background:**

The outbreak of the COVID-19 pandemic sparked numerous studies on policy options for managing public health emergencies, especially regarding how to choose the intensity of prevention and control to maintain a balance between economic development and disease prevention.

**Methods:**

We constructed a cost-benefit model of COVID-19 pandemic prevention and control policies based on an epidemic transmission model. On this basis, numerical simulations were performed for different economies to analyse the dynamic evolution of prevention and control policies. These economies include areas with high control costs, as seen in high-income economies, and areas with relatively low control costs, exhibited in upper-middle-income economies.

**Results:**

The simulation results indicate that, at the outset of the COVID-19 pandemic, both high-and low-cost economies tended to enforce intensive interventions. However, as the virus evolved, particularly in circumstances with relatively rates of reproduction, short incubation periods, short spans of infection and low mortality rates, high-cost economies became inclined to ease restrictions, while low-cost economies took the opposite approach. However, the consideration of additional costs incurred by the non-infected population means that a low-cost economy is likely to lift restrictions as well.

**Conclusions:**

This study concludes that variations in prevention and control policies among nations with varying income levels stem from variances in virus transmission characteristics, economic development, and control costs. This study can help researchers and policymakers better understand the differences in policy choice among various economies as well as the changing trends of dynamic policy choices, thus providing a certain reference value for the policy direction of global public health emergencies.

## Introduction

Since 2020, the COVID-19 pandemic has had a serious impact on countries worldwide. More than three years have passed since the onset of the pandemic. Most economies are still recovering from the effects of COVID-19 and city lockdown [1, 2]. In recent years, there has been an alarming increase in the emergence of new infectious diseases worldwide [3], raising concerns for future public health emergencies. Therefore, the management of such emergencies has become a critical issue.

Various measures have been implemented in response to emergencies such as COVID-19, including isolation from the community, social distancing, reducing crowd, closing school, swift diagnosis, and contact tracing [4–7]. These interventions fall into two categories: the blocking mode and the mitigation mode [8]. The former suggests that some countries have focused on paying short-term costs to avoid significant long-term health and economic losses. The mitigation approach acknowledges the belief held by some policy makers that complete eradication of COVID-19 may not be achievable. While blocking strategies come at a high cost, their impact on the issue is minimal [7].

Numerous scholars have therefore conducted extensive research on the most effective intervention pathways for control policies based on practical experience [9–11]. From an economic perspective, some scholars argue that the optimal control policy depends on the pandemic’s impact on healthcare resources and the populace [7]. Meanwhile, others emphasise that it is a balance between the impact on public health consequences and economic growth [12], or even a trade-off between value of health and the economic cost of implementing control policies [8, 13]. Essentially, a greater number of studies centre on the costs associated with pandemic prevention policies, with a reduced number of studies assessing health benefits. However, when viewed from a health economics standpoint, conducting a cost-benefit analysis is a crucial stage in evaluating the efficiency of public health policy, and the benefits to health constitute integral factors of such analysis.

In addition, existing research has not delved into the issue of the dynamic choice of pandemic control intensity. For example, existing studies have compared the cost-benefit gaps of different control strategies under specific reproduction numbers, or the health and economic outcomes generated by different control strategies under different virus reproduction coefficient scenarios [9]. However, firstly, the virus reproductive coefficients change during an epidemic; secondly, prevention and control policies need to be dynamically adapted to changes in virus characteristics; and thirdly, the costs of implementing prevention and control policies vary widely between economies.

To remedy this, the present study focuses on the dynamic choice model of epidemic prevention policies that include factors such as life-health value, virus transmission characteristics, and economic cost of control policy. Given the complexity of social system, it is hard to estimate the impact of control policy in the short and medium term [14]. Therefore, modelling and simulating the COVID-19 pandemic is a relevant and beneficial way to understand the epidemiological impact of disease transmission and social distance interventions. This model and simulation can provide a theoretical basis and practical experience for the prevention of future major public health emergencies. However, it should be noted that the modelling in this study does not delve into the additional economic losses caused by the COVID-19 pandemic, such as those resulting from business closures and reduced productivity. This is because our cost-benefit model considers the health benefits by measuring economic losses against health losses. To avoid double counting, this is thus not discussed additionally.

This paper is structured as follows. First, we construct a COVID-19 transmission model, including isolation policies. Second, we construct a cost-benefit model for epidemic prevention within a given economy. Third, we simulate the dynamic path of optimal prevention policies based on disease characteristics and control costs. Finally, based on the results obtained, the paper is discussed and concluded.

## Literature review

Research aimed at determining the most effective COVID-19 intervention policy can mainly be classified into two categories: first, measuring the costs of implementing prevention and control policies; second, evaluating the most appropriate intervention policies, and identifying the factors that need to be considered by governments.

Regarding the evaluation of intervention expenses, current research predominantly analyses immediate medical costs such as nucleic acid testing and medical equipment. For example, Bartsch et al. [15] endeavoured to calculate resource consumption and direct medical expenses per symptomatic infection as well as on a national scale to comprehend the potential economic advantages of alleviating the disease burden. The study found that a symptomatic case of COVID-19 could result in a median direct medical cost of $3,045. Another study calculated average treatment costs in Tehran; after evaluating 400 medical records, it was determined that the average treatment cost for COVID-19 was $1,434, with beds and drugs as the most important factors increasing the cost [16]. Another study investigated how COVID-19 affects health and medical costs in China. It then estimated resource use and direct medical costs related to public health [17]. In terms of medical equipment costs, Kapinos et al. [18] compared N95 respirators with surgical masks based on typical surgical operations in the United States to estimate potential savings and avoid healthcare worker (HCW) COVID-19 infections. Their forecast results indicated a reduction of approximately 11 HCW COVID-19 cases per day, and each HCW would need to pay $0.64 to balance the medical system’s profits and losses [18]. These studies offer a practical foundation for policymakers and researchers to investigate the most effective prevention and control policies. Additionally, they serve as a point of reference for this paper’s subsequent numerical simulations’ parameterization.

Regarding the choice of prevention and control policies, a number of scholars have comparatively analysed the advantages and disadvantages of different policies from an economic perspective. These policies include social isolation, nucleic acid testing, lockdowns, and so on. For example. Acemoglu et al. [13] built a multi-group SIR^1^ model to simulate the impaction of different policy types. They found that reducing social interactions, increasing testing scales, and isolating infected populations can minimise economic losses and deaths. Nucleic acid testing has also been found to be a cost-effective alternative to lockdown, making lockdown virtually unnecessary [7]. Berger et al. [19] posited that interventions such as nucleic acid testing (virology testing and serological testing) can facilitate the relaxation of lockdown policies and thus support economic production. A mixed integer nonlinear programming epidemic model has been developed by Biswas and Alfandari [20] to calculate the optimal sequence of non-medical interventions for three different lockdown length scenarios, taking into account the shortage of doctors and hospital beds in France [20].

Of the prevention and control measures mentioned above, lockdown is the most widely used by all countries and the most controversial, because it involves a trade-off between economic freedom and public health. Some studies initially discuss the need for policymakers to consider the impact of transmission rates [21], value of life [7], and implementation time [22] in their policy choices when balancing public health and socioeconomics. In addition, Some scholars have maintained that the ultimate solution to the pandemic is to vaccinate a large number of people to achieve herd immunity. research has expanded the classic SIR model to find the best decision to balance economics and public health during the vaccine promotion process [23]. Consideration has also been given to the age structure of infection cases. Targeted lockdown for different age groups was found to be a useful tool for significantly reducing the number of pandemic deaths and the economic costs of lockdown policies [13, 24].

The research findings present valuable insights for policymakers, but do not discuss the impact of factors such as differences in intervention costs between countries, other characteristics of the disease, the economic loss of health damage, and other factors. Given the diverse contagion characteristics of the disease and varying economic costs, we posit that viral evolution and intervention cost disparities will significantly impact policy decisions. Thus, our analysis focuses on the epidemic lockdown policy and how the optimal approach varies across different control cost levels, from a cost-benefit standpoint.

For the economic impact of health damage, we argue that the pandemic can result in greater economic losses in the absence of non-medical intervention measures. Such loss either stems from self-protective behaviour during the pandemic [25] or from psychological damage, a decreased quality of life, and a decline in work ability due to COVID-19 sequelae even after recovery [26]. For instance, patients who are on the path to recover from COVID-19 usually experience persistent symptoms such as respiratory distress, exhaustion, impaired sensory perception, cognitive issues, chest discomfort, and joint discomfort [27], along with enduring neurological consequences [28]. In addition to this, the COVID-19 pandemic has caused many infection deaths, resulting in a complete loss of life and health for a certain proportion of the population. The human capital element of world economic growth will suffer significant and long-term losses, mainly manifested as labour losses, stagnation of schooling, and the disintegration of global trade and supply chains [29]. Therefore, in the construction of the cost-benefit model of COVID-19 governance, we further incorporate the value of life and health in infected cases and deaths, not only the value of life in fatal cases.

## Methodology

According to existing research, there are various options for COVID-19 prevention and control policies. These include social distancing, tracking exposed populations, nucleic acid or antigen testing, social lockdowns, and isolation of infected populations. Countries around the world choose different policy combinations to manage public health emergencies. The differences in these epidemic control policy combinations ultimately manifest as differences in the intensity of social activity control. Thus, research on the choice of COVID-19 prevention and control policies explores how to optimise the degree of control over social activities. Among the economic models for policy options, the major ones are cost benefit analysis (CBA), cost effectiveness analysis (CEA), cost utility analysis (CUA), social return on investment (SROI), cost consequence analysis (CCA) [30, 31]. Among them, CBA can express costs and benefits in monetary terms, showing decision makers a direct correlation between cost inputs and project outcomes. It is also able to calculate the net benefits to the economy and society as a whole, which can be used to assess whether or not a policy should be implemented in the whole society. Therefore, compared to other economic models, CBA will be more appropriate for the research objectives of this paper.

Our study constructs a cost-benefit analysis framework for the optimal policy based on the theory of epidemic economics and accounting for factors such as the severity of control and control costs. The framework is mainly based on a counterfactual study: The difference in epidemiologic loss of life and cost of disease between policy interventions and no interventions is treated as a benefit of prevention and control, and the medical resources and economic losses of prevention and control are treated as costs. The difference between prevention and control benefits and costs is the net benefit. If the epidemiological characteristics of the virus change (under conditions such as decreased mortality rate and increased infection rate), how would the intensity of control policies evolve to ensure the maximisation of net benefits?

Therefore, we first need to construct a dynamic model of COVID-19 epidemiology based on the epidemiological characteristics of the virus and social activity control policies. We calculate the number of infections, quarantined individuals, exposed individuals, and deaths with non-pharmacological interventions and no interventions, respectively. A cost-benefit model is then established based on these calculations. Finally, by setting different condition ranges, we simulate the intensity of control when the net benefit of the cost-benefit model is maximised to help us analyse the evolution of optimal control policies.

### The SEQIRD model

#### Assumption 1

The populations involved in the transmission include susceptible (S), exposed (E), extra-quarantined (Q), infected (I), deceased (D), and recovered (R) individuals, where (1) the extra quarantined population (Q) mainly refers to the susceptible population who might have been in contact with the identified infected individuals, including sub-close and general contacts. Q refers to those who have a chance of exposure but are not infected. For ease of simulation and calculation, we assume that those in the exposed population who become infected are quarantined for treatment and are not part of the extra-quarantined population. (2) The exposed population refers to those in close contact with the infected population, who will proportionally become infected. Due to the application of big-data-based epidemiological tracing, close contacts can be accurately identified and isolated either by self-isolation or in specific locations. Therefore, even in the absence of strict containment policies, this group bears isolation costs and is not considered part of the extra-quarantined population. (3) The infected population includes asymptomatic individuals, mild cases, and cases of moderate severity and above. (4) The recovered population includes those who recovered after infection and those who received the vaccine from the susceptible population.

**Fig. 1:**
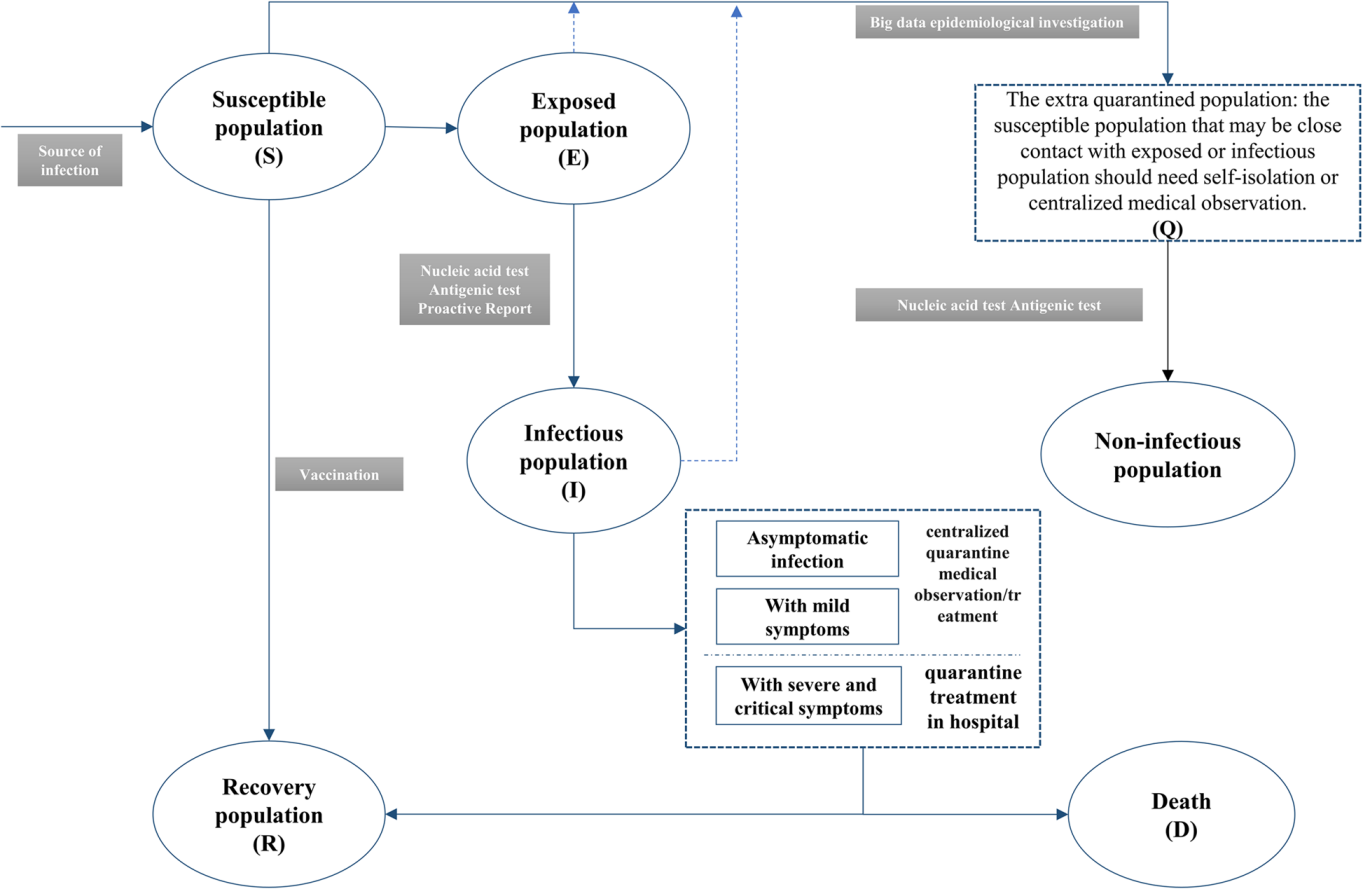
Population dynamics of the SEQIRD model

The composition of the population at a certain period is as follows: ${N}_{t}={S}_{t}+{E}_{t}+{Q}_{t}+{I}_{t}+{R}_{t}+{D}_{t}$. The flow between the different populations is shown in Fig. 1.

#### Assumption 2

According to existing research, current policy types can be divided into two categories based on the intensity of intervention and the degree of strictness of control over social activities: suppression policies aimed at eliminating the virus by lockdown or social distancing, and mitigation policies focused on flattening the curve [7]. Hence, this paper sets the intervention intensity as M, where M is in the range of [0,1]. If policymakers prefer to adopt mitigation policies, then M approaches zero. If policymakers prefer to implement strict control policies, then M approaches one.

#### Assumption 3

The virus transmission rate ${\beta }_{t}$ depends on the virus reproduction number ${R}_{t}$ and the reciprocal of infection duration ${\varvec{\gamma}}$, ${\beta }_{t}={R}_{t}\gamma$ [32]. This involves several parameters: (1) the virus reproduction number ${R}_{0}$ is the basic reproduction number, which usually refers to the natural reproduction rate of the virus without policy control. The time-variant reproduction number ${R}_{t}$ is affected by the strictness of control policies and vaccination. (2) γ is the average rate of recovery or death of the infected population, which is the reciprocal of the infection period.

#### Assumption 4

Changes in the extra quarantined population depend on the quarantine release rate δ. When infection cases are identified, relevant authorities will quarantine susceptible people who may have come into contact with them. These extra quarantined, susceptible individuals can be classified into secondary contacts and general contacts. Close contacts belong to the exposed group. The broader the definition of close contacts in the policy, the higher the proportion of the population that will be quarantined, which may even exceed the infected or exposed population. We assume that the quarantined population comes from the susceptible population, and the population outside quarantine will produce new exposed and infected people based on the increase in the infected population. The main purpose of quarantine is to decrease the rate at which susceptible individuals become exposed and infected by reducing population mobility.

1 $$d{S}_{t}/{d}_{t}=-\beta {S}_{t}{I}_{t}-M*{\lambda }_{I}{E}_{t}$$

2 $$d{E}_{t}/{d}_{t}=\beta {S}_{t}{I}_{t}-{\lambda }_{I}{E}_{t}$$

3 $$d{Q}_{t}/{d}_{t}=M*{\lambda }_{I}{E}_{t} -\delta {Q}_{t}$$

4 $$d{I}_{t}/{d}_{t}={\lambda }_{I}{E}_{t}-{\lambda }_{R}{I}_{t}-{\lambda }_{D}{I}_{t}$$

5 $$dR/{d}_{t}={\lambda }_{R}{I}_{t}$$

6 $$d{D}_{t}/{d}_{t}={\lambda }_{D}{I}_{t}$$

The transmission rate between those six groups depends on virus epidemiological characteristics, control policies, and the degree of social distancing implementation [12]. Changes in the susceptible population ${S}_{t}$ mainly include the emergence of exposed individuals ($\beta {S}_{t}{I}_{t}$), and the extra quarantine population ($M*{\lambda }_{I}{E}_{t}$) created based on the epidemiological investigation of the infected population. ${\lambda }_{I}$ represents the rate of symptoms appearing in the exposed population or the patients who test positive in universal testing (these could be asymptomatic patients, mild cases, or symptomatic individuals).

The severity of control measures directly affects the number of extra quarantined individuals. Under these policies, the direct exposure group consists of close contacts who, if effectively identified, will be isolated. The primary group spreading the virus is the unidentified asymptomatic individuals within the exposed group. However, in stringent control policies, some countries also isolate susceptible individuals who may have been in contact with infected people indirectly. This minimises the chance of the unidentified exposed population spreading the virus. The size of this group typically directly correlates with the number of new infections ${\lambda }_{I}{E}_{t}$, represented as $M*{\lambda }_{I}{E}_{t}$. If the control policy requires a broader range of contacts to be quarantined, such as spatial and temporal companions, the number of quarantined individuals could be one, two, or three times ${\lambda }_{I}{E}_{t}$ depending on the quarantine policy design. Under a policy of complete social relaxation where M = 0, the number of additional quarantined individuals would be zero.

According to the aforementioned epidemic model, this study calculates the cumulative number of infections, quarantines, and deaths over a specific period; this provides a foundation for subsequent simulations and analyses of the cost-benefit of control policies.

7 $$T{E}_{T}={\int }_{0}^{T}\beta {S}_{t}{I}_{t}$$

8 $$T{Q}_{T}={\int }_{0}^{T}M*{\lambda }_{I}{E}_{t}$$

9 $$T{I}_{T}={\int }_{0}^{T}{\lambda }_{I}{E}_{t}$$

10 $$T{D}_{T}={\int }_{0}^{T}{\lambda }_{D}{I}_{t}$$

### The cost-benefit model

The theoretical approach of economic epidemiology provides a fundamental framework for analysing epidemic control policies. In economic epidemiology, the cost-benefit analysis of epidemic control includes two aspects: the costs of disease control and the benefits of disease eradication or control. The results of cost-benefit analysis therefore depend on the difference between policy benefits and costs. Hence, the net benefits $NV$ of control policies can be expressed as the difference between the total benefits $T{B}_{M}$ and total costs $T{C}_{M}$ generated by epidemic control policies: $NV=T{B}_{M}-T{C}_{M}$.

#### Total health and economic benefits of pandemic control policy

The total benefit $T{B}_{M}$ refers to the potential losses avoided by control policies. These include the difference in health economic losses and disease costs of infected individuals under both full freedom of movement and control states. The main reason for considering avoidable potential health economic losses as benefits is that COVID-19 infection not only brings disease costs but also results in economic losses due to the depreciation of human capital (manifested as the reduction of quality-adjusted life years for infected individuals). Different policy choices have differentiated impacts on human capital depreciation; this is also one of the key factors in our analysis of the cost-benefits of COVID-19 governance models.

11 $$\begin{array}{c}T{B}_{M}=\left({TDALY}_{M=0}-TDAL{Y}_{M\in \left(\mathrm{0,1}\right]}\right){Y}_{i}+\left(T{I}_{M=0}-T{I}_{M\in \left(\mathrm{0,1}\right]}\right){C}_{Ei}\\ ={\left(YLDs*T{R}_{t}+YLLs*T{D}_{t}\right)}_{M=0}{Y}_{i}-{\left(YLDs*T{R}_{t}+YLLs*T{D}_{t}\right)}_{M\in \left(\mathrm{0,1}\right]}{Y}_{i}+\left(T{I}_{M=0}-T{I}_{M\in \left(\mathrm{0,1}\right]}\right){C}_{Ei},\end{array}$$

Where TDALY refers to the years of life lost due to death and illness from COVID-19 [33]. TDALY is an increasing function of infection rate and infection mortality rate. The measurement unit is disability-adjusted life years (DALYs),^2^ which can be expressed as the sum of years of life lost (YLLs) and years lost due to disability (YLDs) among infected individuals [10].

${\left(YLDs(T{I}_{t}-T{D}_{t})+YLLs*T{D}_{t}\right)}_{M=0}{Y}_{i}$ represents the health economic loss of the infected population under a state of complete non-control; ${\left(YLDs(T{I}_{t}-T{D}_{t})+YLLs*T{D}_{t}\right)}_{M\in (\mathrm{0,1}]}{Y}_{i}$ represents the health economic loss of all DALYs in the infected population under some degree of control; ${Y}_{i}$ is the health economic loss per person per DALY - the economic output corresponding to a year of life.^3^

$\left(T{I}_{M=0}-T{I}_{M\in \left(\mathrm{0,1}\right]}\right){C}_{Ei}$ represents the epidemiological cost that can be avoided after the implementation of control policies. Generally, the costs associated with an epidemic contain both epidemiological costs and excess burden. Epidemiological costs refer to the cost of treatment, lost wages, and physical and mental suffering of the infected population. Excess burden refers to the costs related to disease prevention, such as self-protection and vaccination costs. Regarding excess burden, as long as the epidemic exists, regardless of control, vaccine costs and self-isolation costs will exist for each person. Therefore, in this formula, ${C}_{Ei}$ only represents epidemiological costs. It should be noted that the infected population includes not only patients with different degrees of infection severity but also those who died after infection. Here, we do not distinguish the structure of epidemiological costs among the infected population.

#### Total intervention cost of pandemic control policy

The intervention cost of control policy (${\varvec{T}}{{\varvec{C}}}_{{\varvec{M}}}$) includes direct control costs and indirect costs brought about by control. Direct costs refer to the medical and social resources required for control social activity, such as additional isolation sites, medical staff, testing costs, medical observation, and so on. Indirect costs refer to the economic slowdown or stagnation brought about by control, such as economic losses caused by people being restricted from conducting productive activities. The expression is as follows:

12 $$T{C}_{M}=\left({C}_{Qi}+{C}_{Wi}\right)T{Q}_{M\in \left(\mathrm{0,1}\right]}*\mathrm{Days},$$

where $T{Q}_{M\in \left(\mathrm{0,1}\right]}$ is the total number of people quarantined during the pandemic, ${C}_{Qi}$ is the per capita direct cost due to control policy, ${C}_{Wi}$ is the per capita indirect cost, and Days refers to work time loss because of quarantine.

#### Net value of pandemic control policy

13 $$\underset{M}{\mathrm{max}}\, NV=T{B}_{M}-T{C}_{M}$$

14 $$={\left(YLDs*T{R}_{t}+YLLs*T{D}_{t}\right)}_{M=0}{Y}_{i}-{\left(YLDs*T{R}_{t}+YLLs*T{D}_{t}\right)}_{M\in \left(\mathrm{0,1}\right]}{Y}_{\begin{array}{c}i\\ \end{array}}+\left(T{I}_{M=0}-T{I}_{M\in \left(\mathrm{0,1}\right]}\right){C}_{Ei}-({C}_{Qi}+{C}_{Wi})T{Q}_{M\in \left(\mathrm{0,1}\right]}*\mathrm{Days}$$

s.t. Eqs. (1)–(10)

Based on Eqs. (7)–(13), Eq. (14) can be derived, in which the net benefit is expressed as a function of parameters such as infection rate, mortality rate, number of exposed people, and others. Cost estimation can be done without considering discounting. Given the relatively short timeframe of the pandemic outbreak, a social discount rate between 3 and 5% makes little difference within a year [34].

In accordance with the principle of maximising net value, we use the GEKKO proposed by Beal et al. [35] to conduct numerical simulations to solve for the optimised M when maximising the net benefit.

### Parameter setting

For the above SEQIRD model and cost-benefit model, this paper uses the IPOPT solver in GEKKO to calculate the optimal solution for linear programming [35]. For the parameters of the SEQIRD model, we refer to the research of Berger et al. [19], mainly adopting conclusions that are as consistent as possible in most studies. Of course, there are multiple variants of the coronavirus, so its epidemiological characteristics will change with each variation. To account for this, we categorise the virus variants into three major types: the initial outbreak of COVID-19, the Delta variant series, and the Omicron variant series. There are significant characteristic differences between these three series.

In addition, for the parameters of the cost-benefit model, different countries face different cost levels, which is one of the key factors driving various economic entities to adopt different control policies. For this, we divide the economic entities into high-cost and low-cost economies. The high-cost economy mainly includes high-income economic entities, while the low-cost economy mainly includes middle- and low-income economic entities.

We have summarised and collated the parameters estimated and measured in the existing literature and set the model parameters in this article based on these. The definitions and numerical ranges of the parameters in the model are as follows:

1. Basic reproduction number $${{\varvec{R}}}_{0}$$: according to existing research, we set the range of $${{R}}_{0}$$ to be between 1.4 and 24. This is mainly based on the large number of estimates for the basic reproduction number of the coronavirus made by scholars during the early outbreak in 2020. For example, some scholars estimated the average $${{R}}_{0}$$ in countries like the US and Japan to be between 3 and 5 [36]. Estimates for $${{R}}_{0}$$ during the COVID-19 pandemic in Wuhan, China, range from 3.11 to 6.47 [37–39], or 1.4 to 6.47 [40]. With the virus mutations, some scholars have estimated the transmission characteristics of the Omicron variant in five countries including India, Indonesia, Malaysia, Bangladesh, and Myanmar, and found that the range of reproduction number is between 0 and 9 [41]. The range of basic reproduction number for each series of viruses is shown in Table 1.
2. Infection rate $${{\varvec{\lambda}}}_{{\varvec{I}}}$$**:** the infection rate can be defined as the proportion of the exposed population that becomes infected, which is equal to the reciprocal of the average incubation period. Regarding the infection rate of the virus during the uncontrolled period in the early days in Wuhan, some studies found that the range of $${\lambda }_{I}$$ is between 1/5 [28] and 1/3 [42]. With the mutation of the virus, the average combined incubation period is 6.57 days, and the average incubation days vary among different variants. For example, the average incubation period for cases caused by the Alpha variant is 5.00 days, 4.50 days on average for the Beta variant, 4.41 days on average for the Delta variant, and 3.42 days on average for the Omicron variant [43]. Some research has indicated that the incubation period for Delta is 4.16 ± 2.03 days, 4.85 ± 2.37 days for Omicron BA.1, and 4.17 ± 1.94 days for Omicron BA.2 [44]. Accordingly, the infection rate of the virus is the reciprocal of the above incubation days. The range of incubation periods for virus is detailed in Table 1.
3. Reciprocal of infection duration $${\varvec{\gamma}}$$**:** this is the proportion of the infected population that becomes the recovered and dead population, represented as the reciprocal of the average duration of disease. Existing research estimates the range of the reciprocal of disease duration to be between 1/18 [28] and 1/5 [42]. When the infection incubation period is around 5 days, the duration of the disease is correspondingly set at 18 days [28]. The range of disease duration is detailed in Table 1.
4. Infection fatality rate $${{\varvec{\lambda}}}_{{\varvec{D}}}$$**: **there are two kinds of measures for infectious disease fatality rate, including case fatality ration (CFR) and infection fatality ratio (IFR) [45, 46]. When all infection cases can be fully identified, the value of CFR and IFR would be the same; otherwise, CFR will overestimate IFR. Some scholars have estimated IFR, finding that the IFR of the early virus is approximately between 0.5% and 1% [45, 46]. The infection fatality rate of the Omicron variant in India, Indonesia, Malaysia, Bangladesh, and Myanmar is estimated to be between 0.016% and 0.136% [41]. The range of IFR is detailed in Table 1.
5. Recovery rate $${{\varvec{\lambda}}}_{{\varvec{R}}}$$**:** the recovery rate can be expressed as $${{\lambda}}_{{R}}=\gamma -{\lambda }_{D}$$ because the population faces two scenarios after a certain course of the disease: recovery or death.
6. Rate of release from quarantine $${\varvec{\delta}}$$**:** since the virus incubation period is between 5 and 18 days, during the early outbreak of COVID-19 in Wuhan, the isolation period was 14 days. In this study, the contact isolation ratio during the containment period is calculated as 1/14.
7. Disability-adjusted life years $${\varvec{D}}{\varvec{A}}{\varvec{L}}{\varvec{Y}}{\varvec{s}}$$: according to existing research, different degrees of disease symptoms cause different losses of life. According to [22], the YLLs caused by COVID-19 would be 14.24 years per case. YLDs are summed for mild, severe, and critical illnesses. To compare the results more intuitively in this paper, we calculated the weighted average YLDs for the above three symptoms according to the parameters in table 1 of Zhao et al. [22], namely$$\sum proportion\, of\, cas{e}_{n}*disability\, weigh{t}_{n}*duration\, of\, cas{e}_{n}=0.815*0.01*0.04+\left(0.138+0.047\right)*0.53*0.12=0.01$$.^4^ Furthermore, some research has indicated that COVID-19 may leave certain sequelae, and the life years loss caused by sequelae of the disease per person in Zhao et al. [22] research is $$\sum disability\, weigh{t}_{n}*duration\, of\, cas{e}_{n}=0.17*0.25=0.0425$$. Therefore, the YLDs are set as 0.0525 per case in the simulation data.
8. Per capita output $${{\varvec{Y}}}_{{\varvec{i}}}$$**:** this indicator refers to the economic loss of disability-adjusted life years, measured here by gross domestic product (GDP) per capita [22]. Since the measurement of the value of life is more controversial as well as influenced by different values, this paper only analyses the economic loss due to health loss from the economic perspective, which also reflects the relative evaluation of the value of life. Different economies have different GDP per capita, which can be classified as high income, upper-middle income, lower-middle income, and low income according to World Bank statistics. The global average per capita GDP was $12,236 in 2021. This study selects high-income economies and upper-middle-income economies both above and below this average as the two typical objects for comparison in the cost-benefit model.
9. Per capita epidemic cost $${\varvec{C}}_{\varvec{Ei}}$$**:**$${C}_{Ei}$$ is defined as the direct medical costs per case. Wage loss due to illness is already expressed in $${Y}_{i}$$, so it is not double-counted here. High-income economies usually have different direct medical costs of epidemics with other income-level economies, i.e. upper-middle income, low income economies. For instance, existing research suggests that the range of average medical costs for different COVID-19 symptoms (general care, inpatient care, critical care patients) in the United States is approximately $9,763–$61,168 per case [47], with a national average medical cost $3,045 per case [15]. For other upper-middle-income economies like China, the weighted treatment cost for severe, moderate, and mild confirmed COVID-19 cases is ¥22,061.94 (USD $3,192.76) per case [48].
10. Direct cost per capita of quarantine $${\varvec{C}}_{\varvec{Qi}}$$**: **For upper-middle-income economy, existing research by Jin et al. [48] found that the average direct medical cost of managing the quarantined population is ¥584.08 (USD $84.53) per person, which includes case identification, testing, and medical observation during isolation. The direct non-medical cost includes fees for isolation sites. The per capita isolation cost for those testing negative is ¥150 (USD $21.4) per person per day [48]. Therefore, the per capita direct cost of isolation is the sum of the direct medical cost and the direct non-medical cost, averaging ¥734.08 (USD $104.87) per person per day. In high-income countries like the United States, the direct non-medical cost of managing the isolated population in alternative care sites is $304 per person per day^5^ [49], and the testing cost is $51 [49], which totals $355.
11. Indirect cost per capita of quarantine $${\varvec{C}}_{\varvec{Wi}}$$: there is a significant difference in indirect costs between high-income and lower-middle-income countries. We use the average daily wage per capita to express the indirect losses of isolated personnel. It is important to note that the indirect losses experienced by isolated individuals may differ depending on their occupation and income level.
12. Quarantine duration per capita $${\varvec{D}}{\varvec{a}}{\varvec{y}}{\varvec{s}}$$**:** the number of days of quarantine varies at different stages of the epidemic. The government mandates an average of 14 days of isolation for quarantined people [48].

**Table 1:** Parameter settings of the SEQIR model and cost-benefit model

**Panel A**				
	COVID-19	Delta	Omicron	Range
$${{\varvec{R}}}_{0}$$	1.4~6.49 [40] 3 [50]	5.0~7.23 [51] 6.4 [50]	0~9 [41] 5.5~24 [52] 9.5 [50]	1.4~24
$$1/{{\varvec{\lambda}}}_{{\varvec{I}}}$$**(day)**	3~5 [28, 42] 5 [53] 6.8 [50]	2.13~6.19 [44] 3.9~5 [51] 3.7 [53] 5.8 [50]	BA.1: 2.48~7.22 BA.2:2.23~6.11 [44] 3 [50]	2~7.22
$$1/{\varvec{\gamma}}$$(day)	5~18 [28, 42]	8~10^a^ 11.3 [50]	11 [50]	5~18
$${{\varvec{\lambda}}}_{{\varvec{D}}}$$	0.005~0.01 [45, 46]	0.00071~0.002 [54]	0.00016~0.00136 [41]	0.0001~0.01
**Panel B**				
**Group**	High control cost economy (High-income economy)		Low control cost economy (Upper-middle-income economy)	
$${Y}_{i}$$	USD$ 48,225.2^b^ (RMB: 33,7576)		USD $10,828^c^ (RMB: 75,796)	
$${C}_{Ei}$$	USD$3,045 (RMB:21,315) [15]		USD $3,192.76 (RMB: 22,061.9) [48]	
$${C}_{Wi}$$	USD $240/per day^d^ (RMB:1680)		¥272/per day [22]	
$${C}_{Qi}$$	USD $355/per day [49] (RMB:2485)		¥734.08/per day [48]	

The exchange rate is calculated at 7 RMB per USD. The per capita epidemic cost, the per capita indirect cost of isolation, and the per capita direct cost of isolation mainly refer to the United States and China. This is only used to indicate cost differences across economies

^a^https://theconversation.com/how-contagious-is-delta-how-long-are-you-infectious-is-it-more-deadly-a-quick-guide-to-the-latest-science-165538

^b^The source of the data is the average GDP per capita value of high-income countries in 2021 published by the World Bank. https://data.worldbank.org.cn/indicator/NY.GDP.PCAP.CD?locations=XD&most_recent_value_desc=true

^c^The source of the data is the average GDP per capita value of upper-middle-income countries in 2021 published by the World Bank.https://data.worldbank.org.cn/indicator/NY.GDP.PCAP.CD?locations=XO

^d^According to the average hourly wage data for 2021 published by the U.S. Bureau of Labor Statistics, the rate is approximately $30 per hour. In this study, we calculate daily wages based on an 8-h workday. https://www.bls.gov/ces/data/employment-and-earnings/2021/summarytable_202112.htm

COVID-19 has gone through variants like D614G, Beta, Delta, and Omicron. We list the features of virus strains at different periods below according to existing studies (see Table 1), such as reproduction number, incubation period, duration of illness (proportion corresponding to the transition to the recovered and deceased population), and infection fatality rate.

In accordance with Panel A of Table 1, the initial virus had less ability to spread relative to mutant strains such as Delta and Omicron, but the mortality rate was much higher than that of other mutant strains; this is due to both the decrease in lethality during the mutation of the virus itself and the implementation of vaccination programmes in various countries. The trends in the evolution of epidemiological characteristics can be summarised as follows. Firstly, the basic reproduction number increases with virus variation and the incubation days become relatively shorter, which indicates that the infection rate is increasing with variation and the transmission rate is also increasing. Secondly, the disease duration gradually decreases. Thirdly, the mortality rate of infection decreases with variation, vaccine, and treatment.

In addition, various economies face different cost levels for prevention and control, especially for economies with different income levels. In this regard, this paper establishes two sets of initial values (see Panel B): One group consists of high-income economies with high COVID-19 epidemic prevention and control costs, represented by the United States in Panel B; the other group consists of upper-middle-income economies with low COVID-19 epidemic prevention and control costs, represented by China in Panel B. We investigate how the optimal intensity of COVID-19 control policies in economies with different cost levels changes with the evolution of virus transmission characteristics.

All economies face changes in virus transmission characteristics. We limit these changes to two scenarios. The first is based on the initial virus transmission characteristics of relatively low reproduction numbers, a long incubation period, a long infection period, and a high mortality rate. The second scenario is based on the transmission characteristics of variants like Delta and Omicron. This scenario is characterised by relatively high reproduction numbers, a short incubation period, a short infection period, and a low mortality rate.

For the first scenario (Scenario 1), which has relatively low reproduction numbers, a long incubation period, a long infection period, and a high mortality rate, we set the initial values as follows: a basic reproduction number of 3, an incubation period of 6.5 days, an infection period of 18 days, and a mortality rate of 0.01. Based on this, while keeping other parameters constant, we adjust one parameter within the variable range set by Table 1. We then analyse how the degree of pandemic control in different economies changes with that parameter.

For the second scenario (Scenario 2), characterised by relatively high reproduction numbers, a short incubation period, a short infection period, and a low mortality rate, we set the initial values as follows: a basic reproduction number of 9.5, an incubation period of 3 days, an infection period of 11 days, and a mortality rate of 0.0001. Similarly, while keeping other parameters constant, we adjust one parameter within the variable range set by Table 1. We then analyse how the degree of pandemic control in different economies changes with that parameter.

## Results

### $M$ and basic reproduction number

#### Scenario 1 with transmission characteristics of initial virus

**Fig. 2:**
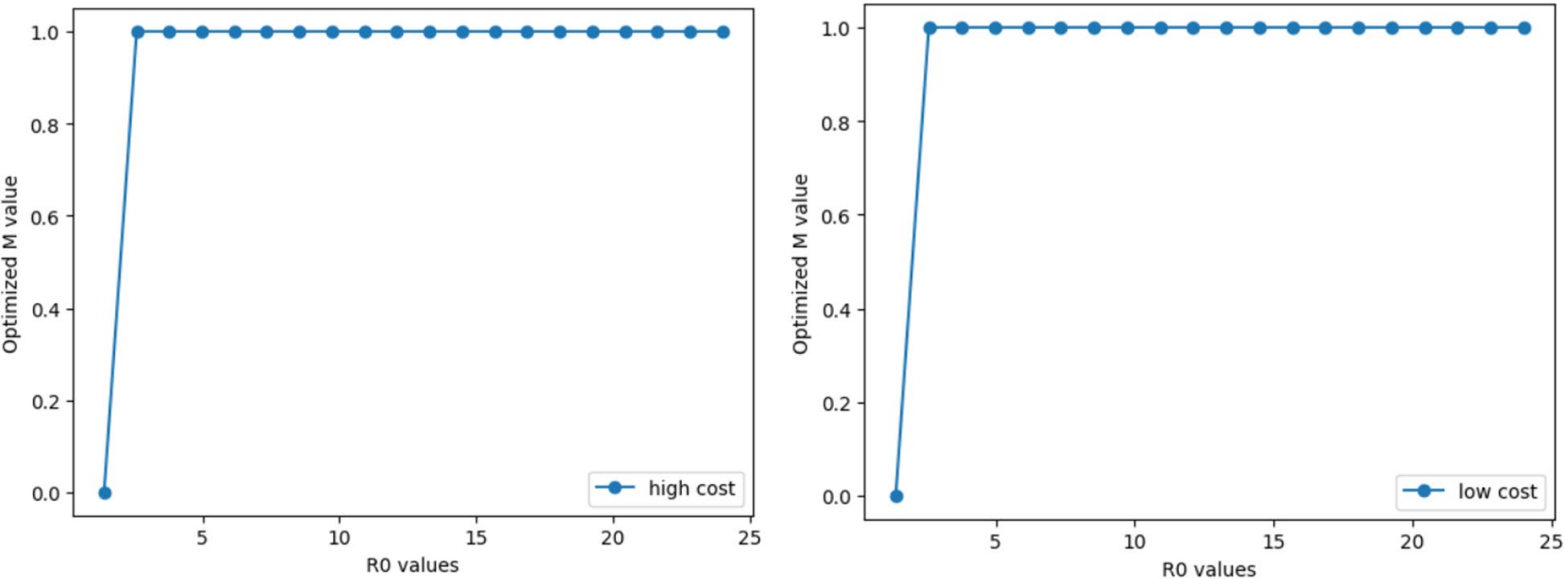
The trend of M’s optimal value with R0 for scenario 1

Keeping parameters such as the long incubation period, long infection period, and high infection fatality rate constant, we randomly select 20 points within the range of 1.4 ~ 24 for the basic reproduction number ${R}_{0}$; this is done to simulate the evolution of the basic reproduction number. Based on this, we use GEKKO to simulate and solve the dynamic path of COVID-19 transmission under the SEQIRD model; this is to solve for the M value at the time of cost-benefit model optimisation under a specific ${R}_{0}$ value. As shown in Fig. 2, the results support that for both high-cost and low-cost economies, when facing a virus with long incubation, long infection period, and high mortality, the optimal control policy is $M=0$ (complete release) only when the basic reproduction number is very small. Apart from this, regardless of how ${R}_{0}$ evolves, $M=1$ (strict control) is the optimal choice for these two types of economies; this also means that for the initial transmission characteristics of COVID-19, the optimal policy choice for relevant governments is strict control.

#### Scenario 2 with transmission characteristics of variants

**Fig. 3:**
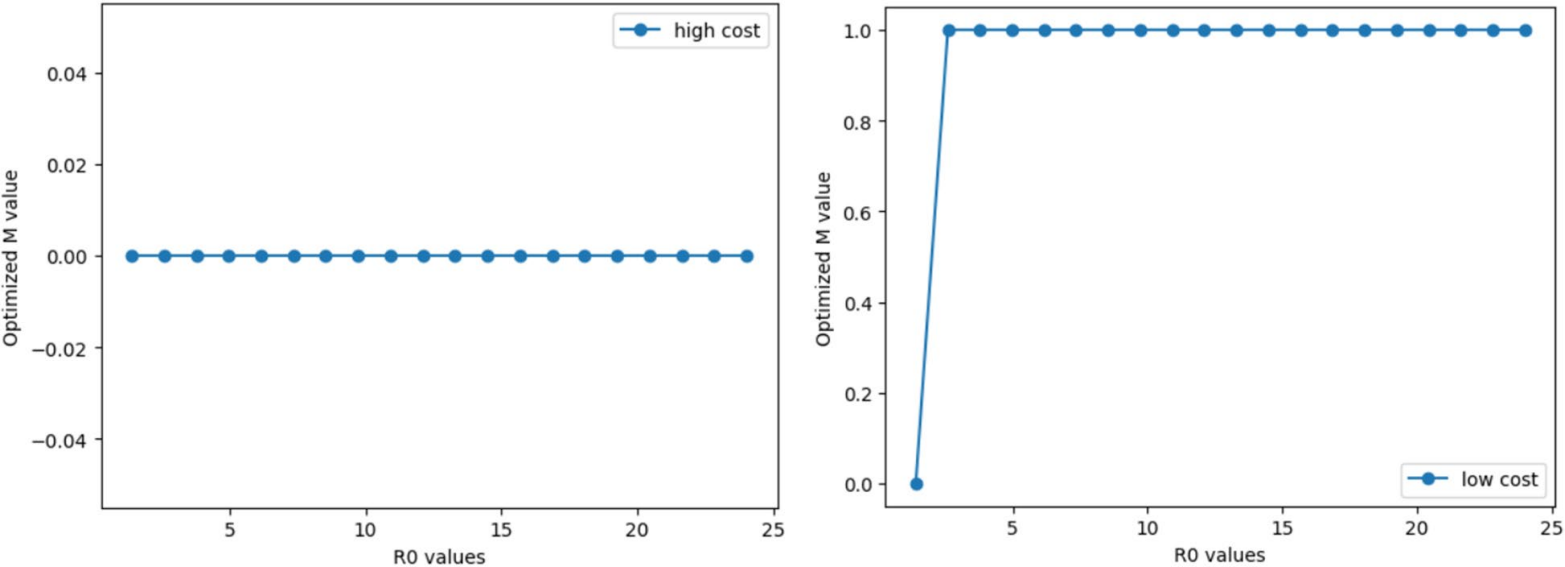
The trend of M’s optimal value with R0 for scenario 2

Keeping parameters such as short incubation period, short infection period, and low infection fatality rate constant, we also select 20 points uniformly at random within the range of 1.4 ~ 24 for the basic reproduction number. Through GEKKO simulation to solve the SEQIRD and cost-benefit models, the results indicate that high-cost and low-cost economies present two extremely different optimal control strategies (see Fig. 3 for details). For high-cost economies under conditions of short incubation, short disease course, and low mortality, the optimal choice of control policy remains $M=0$ with the evolution of the basic reproduction number ${R}_{0}$. However, the optimal choice for low-cost economies is to choose the strictest control, that is, $M=1$, when ${R}_{0}> 1.4$. This indicates that as the virus becomes more infectious but the mortality rate decreases, strict control policies lose their cost-effectiveness in high-cost economies, though they can still maintain a positive net income in low-cost economies.

### $M$ and incubation period

#### Scenario 1 with transmission characteristics of initial virus

**Fig. 4:**
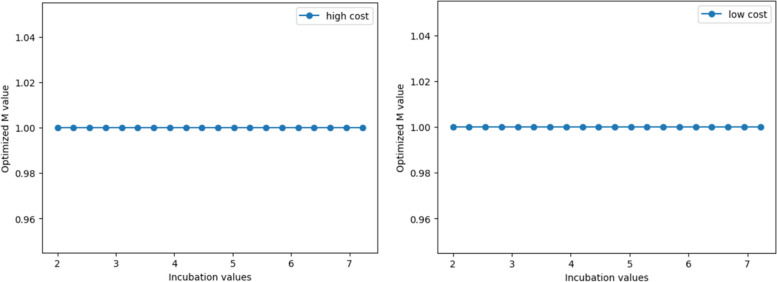
The trend of optimal M values with incubation values for scenario 1

Keeping the parameters of a relatively low basic reproduction number, a long infection period, and a high mortality rate constant, we adjust the incubation period. We simulate the SEQIRD model and cost-benefit model to find the optimal control policy for different incubation periods. The results demonstrate that the strictest control policy is the optimal choice for both high- and low-cost economies (Fig. 4). This indicates that for infectious diseases with a high mortality rate, a long infection period, and a certain reproduction number, strict control in the early stages of the outbreak can maximise the benefits of epidemic control and avoid more loss of life.

#### Scenario 2 with transmission characteristics of variants

**Fig. 5:**
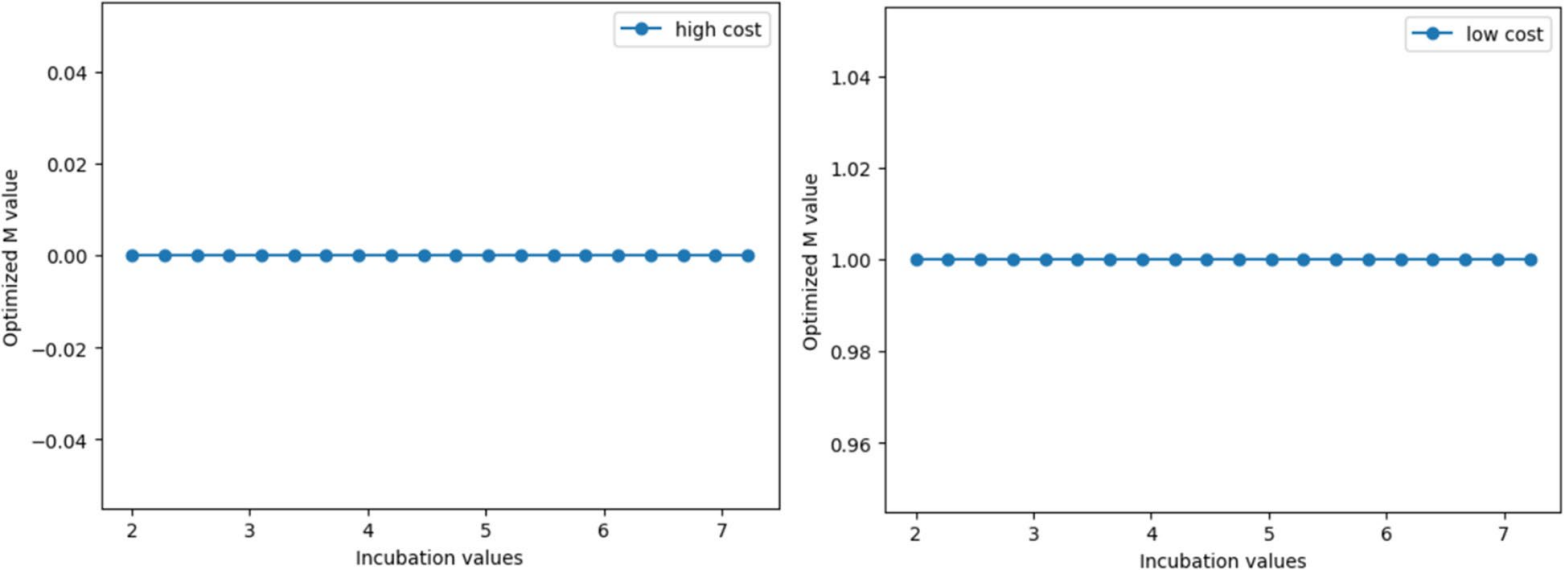
The trend of optimal M values with incubation values for scenario 2

When maintaining parameters of relatively high basic reproduction number, short disease duration, and low infection fatality rate, as the incubation period changes from 2 days to 7.22 days, the optimal control intensity choices of economies at different cost levels have different results compared to Scenario 1. The results in Fig. 5 show that for high-cost economies facing high basic reproduction numbers, short disease durations, and low mortality rates, the optimal control policy is no mandatory quarantine and complete liberalisation of social activities even if the virus incubation period changes. For low-cost economies, maintaining strict control can still achieve the optimal net benefit.

### $M$ and infection duration

#### Scenario 1 with transmission characteristics of initial virus

**Fig. 6:**
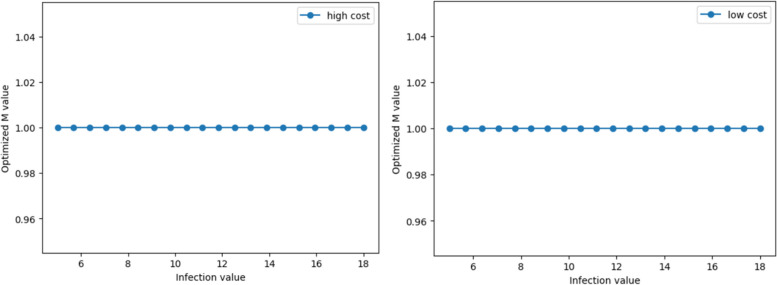
The trend of optimal M values with infection duration for scenario 1

While keeping the parameters of a relatively low basic reproduction number, a long incubation period, and a high mortality rate constant, we change the infection duration of COVID-19. The range of change is 5 to 18 days. For both high and low cost economies, the simulation results suggest that choosing the strictest control policy is the optimal choice for both types of economies (Fig. 6). In the early stages of the COVID-19 outbreak, the virus characteristics met the criteria of a relatively low basic reproduction number, a long incubation period, a high mortality rate, and a relatively long disease duration. Accordingly, all national economies choose strict controls to prevent a widespread outbreak of COVID-19 and avoid mass deaths.

#### Scenario 2 with transmission characteristics of variants

**Fig. 7:**
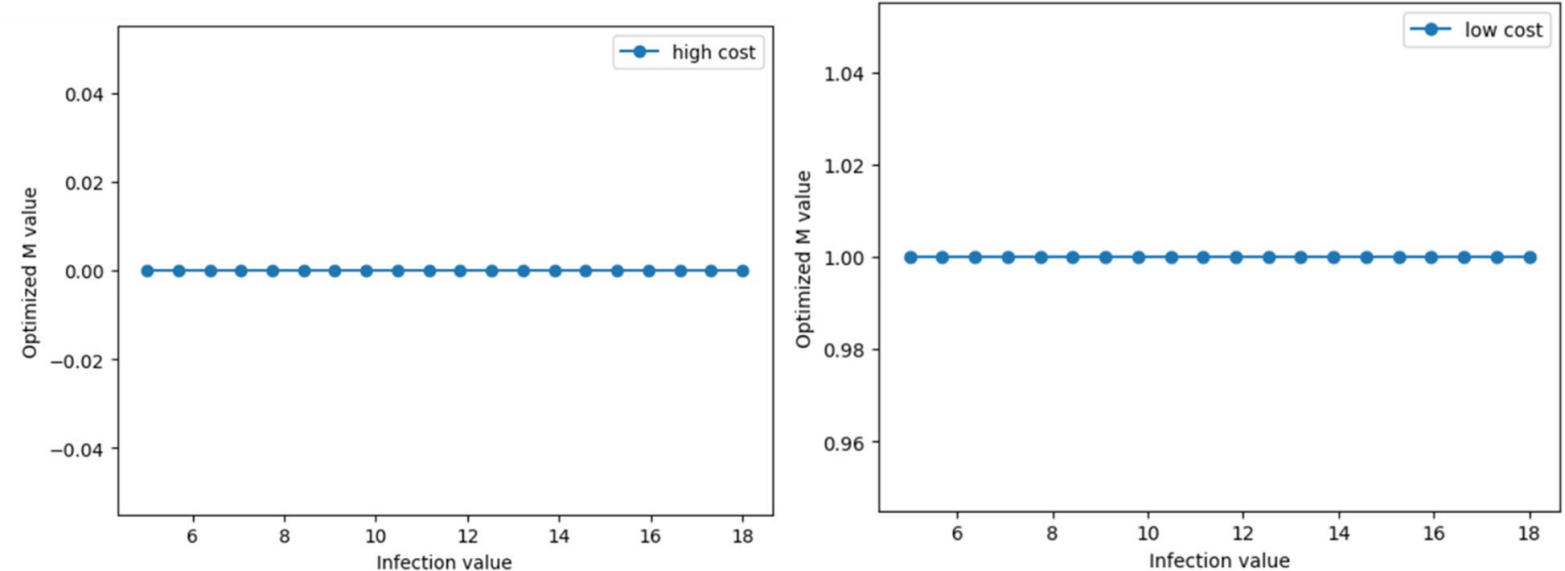
The trend of optimal M values with infection duration for scenario 2

Maintaining the parameters of relatively high basic reproduction number, short incubation period, and low mortality rate, we change the infection duration of COVID-19 for dynamic simulation of the SEQIRD and cost-benefit models. Similar to the results of Scenario 2 with transmission characteristics of variants of section M and incubation period, high-cost economies in this scenario are more likely to choose a completely non-isolation policy (Fig. 7). We believe this may be because the IFR of COVID-19 has dropped significantly, making the net benefit of strict control unable to cover the cost of quarantining impacted individuals. However, for low-cost economies, the cost of control is lower. The net benefit of strict control can still cover the control costs, and thus the simulation results lean more towards strict control.

### $M$ and infection fatality rate

#### Scenario 1 with transmission characteristics of initial virus

**Fig. 8:**
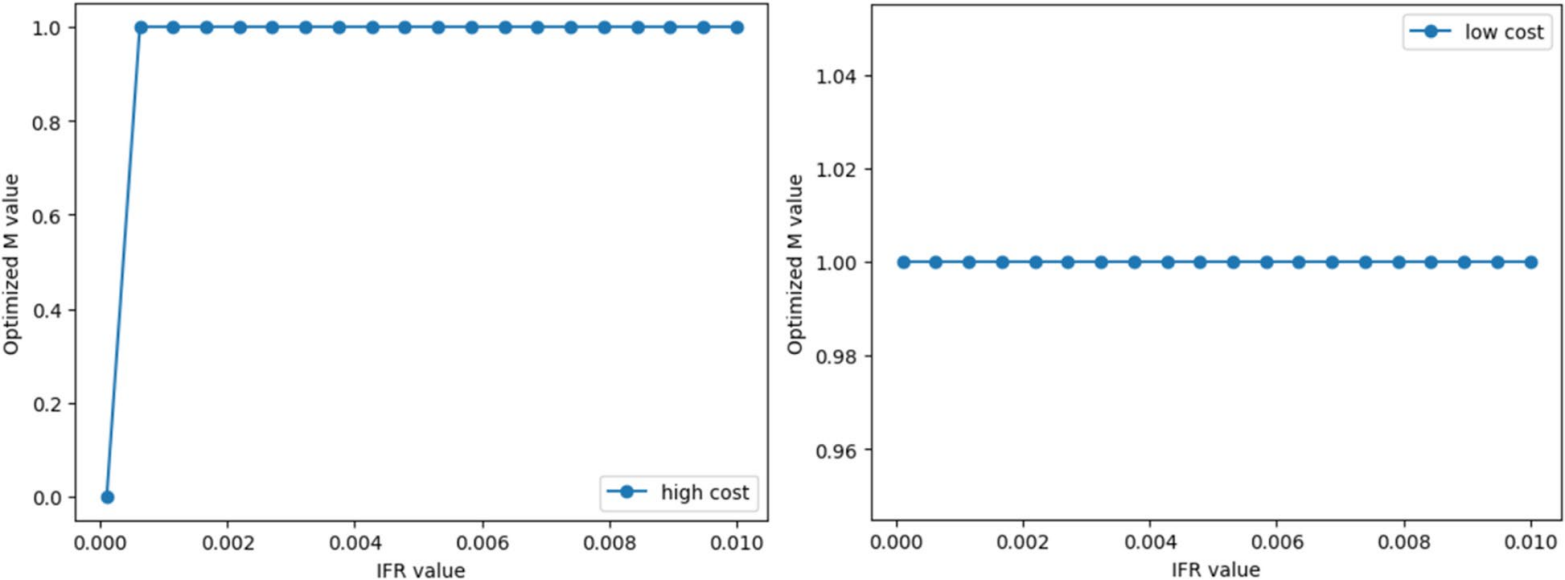
The trend of optimal M values with infection fatality rates for scenario 1

Keeping the parameters of a relatively low basic reproduction number, a long incubation period, and a long infection period constant, we change the infection fatality rate of COVID-19. The range of change is from 0.0001 to 0.01. Dynamic simulation results indicate that when the infection mortality rate exceeds a certain threshold, the control policies of high-cost economy tend to be the strictest (Fig. 8). The strategy of low-cost economy always leans toward the strictest control.

#### Scenario 2 with transmission characteristics of variants

**Fig. 9:**
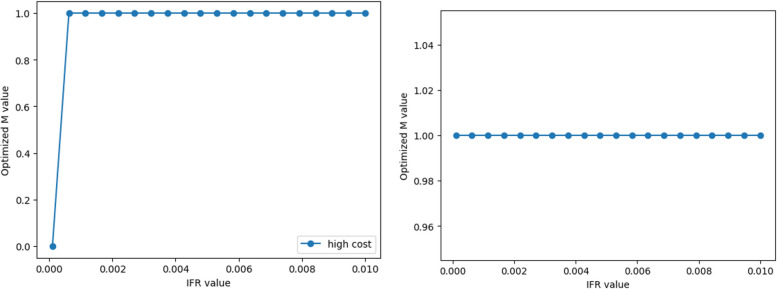
The trend of optimal M values with infection fatality rates for scenario 2

When faced with a scenario of a relatively high basic reproduction number, a short incubation period, and a short infection period, dynamic simulation results reflect a similar policy choice tendency for high-cost economy to Scenario 1. Low-cost economy also tends to adopt the strictest control strategies, suggesting that changes in mortality rates are key factors affecting control costs and strategies in different economies (Fig. 9).

### Adjusted model incorporating extra economic costs of control policy

The results of the ***M*** and basic reproduction number, ***M*** and incubation period, ***M*** and infection duration and ***M*** and infection fatality rate sections evidence that, under the premise of maximising net value, the control policy of low-cost economy tends to favour strict control in all scenarios except when the basic reproduction number is particularly low. The main reason for this result is that the existing cost-benefit model only considers the dead, infected, and quarantined populations but does not yet consider extra burdens on the remaining susceptible population. These burdens include the extra burden of self-protection and the negative impact of economic stagnation caused by isolation and strict control. Decision-making in low-cost economy would change if these extra costs were factored into the cost-benefit model. We further discuss this matter in this section.

In constructing the cost-benefit model, if we further consider the economic losses incurred by the remaining susceptible population due to control policies, such as business closures, unemployment, and other economic stagnation [55], we should add an extra cost parameter ${C}_{extra}$ to Eq. (13). This parameter is influenced by a variety of factors such as the value of M, the duration of M ($T$), macroeconomic development (GDP), and economic structure (Structure). Different economic structures and developments will result in different social resilience, and therefore the extra economic loss caused by control policies will also vary. Consequently, we obtain the new Eq. (15) for net value.

15 $$\underset{M}{\mathrm{max}}\, N{V}_{2}=NV-{C}_{extra}(M,GDP,Structure,T)$$

s.t. Eqs. (1)–(10)

It is assumed that ${C}_{extra}$ is an increasing function of M. This assumption is largely based on real-world performance. As the severity and duration of the lockdown increases, the additional economic losses are compounded by greater restrictions on population mobility, lower levels of economic activity and increased restrictions on trade flows. For example, one study found that a one-month total lockdown of a city would halve intercity truck traffic in China. A one-month blockade of a major city such as Shanghai would result in a 10 per cent drop in national real income [56]. However, society still faces economic losses during the pandemic, mainly in terms of the economic losses caused by health life loss in Eq. (14) when the M = 0, i.e. ${\left(YLDs*T{R}_{t}+YLLs*T{D}_{t}\right)}_{M=0}{Y}_{i}$, while ${C}_{extra}=0$. Thus, the optimal choice of M depends on the relationship between $NV$ and ${C}_{extra}(M,GDP,Structure,T)$.

**Fig. 10:**
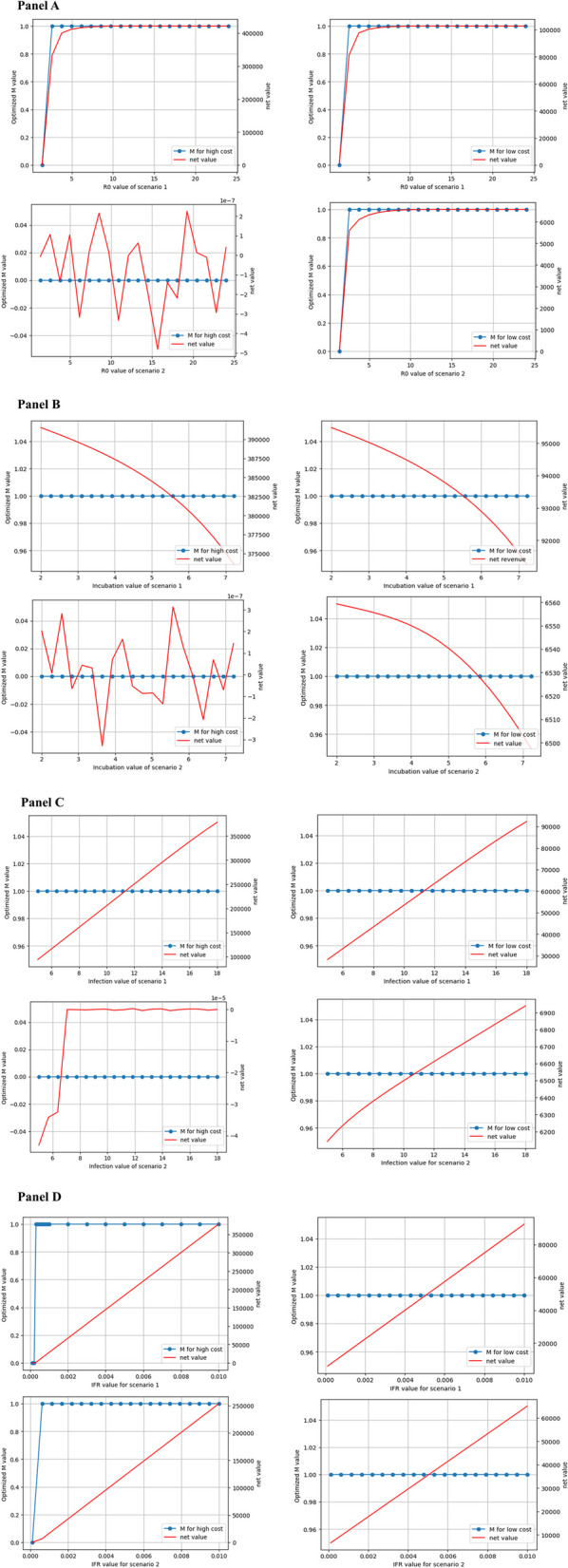
The optimal M values and their corresponding net value (net value of all subgraphs in RMB)

Based on the optimisation calculations in Results section, we obtained the net value corresponding to each optimal M value as shown in Fig. 10. Since the population numbers obtained from the SEQIRD model are all represented by proportions, the net value obtained from the cost-benefit model is the weighted net value per capita. Figure 10, when M = 0, the net income of all economies is close to 0. If ${C}_{extra}=0$, thus $N{V}_{2}$ is also close to 0. However, when M = 1, the net value of all economies is positive in Fig. 10. If ${C}_{extra}$ is greater than NV when M = 1, then $N{V}_{2}$ is less than zero, or less than the value when M = 0. As such, M = 1 will no longer be the optimal choice for those economies.

Taking ***M*** and basic reproduction number section as an example, regardless of scenario 1 or scenario 2, the net value of the optimal control policy of the low-cost economy is positive. In scenario 1, NV is around RMB ¥100,000 per capita, and in scenario 2, NV is less than RMB ¥7,000 per capita, as shown in Panel A of Fig. 10. If the ${C}_{extra}$ caused by strict control policies is greater than the aforementioned net value, the strict control of the low-cost economy will lose its cost-effectiveness. At this point, the optimal policy needs to return to complete relaxation to achieve the maximisation of net value.

Looking back at the policy practises of various countries during the pandemic, as strict control policy continues, unemployment rates and trade stagnation will continue to rise, and various businesses will face further problems such as capital chain and supply chain disruptions. Although the government will introduce corresponding market rescue policies, national funds are limited, and they cannot cover every group, which may exacerbate inequality.

## Discussion

Based on the simulation results in ***M*** and basic reproduction number, ***M*** and incubation period, ***M*** and infection duration, ***M*** and infection fatality rate and Adjusted model incorporating extra economic costs of control policy sections, we find the following aspects worthy of further discussion.

1. For the initial outbreak of the COVID-19 virus (scenario 1), which is characterised by a relatively low reproduction number, a long incubation period, an extended infection period, and a high fatality rate, both high-cost and low-cost economies have a reason to implement the most stringent lockdown to maximise net benefits. In practice, countries did adopt stricter precautionary measures in the early stages of the COVID-19 pandemic. The term “early” mainly refers to the outbreak of COVID-19 in during 2020. To illustrate, Porcher’s [57] national policy statistics reveal that the COVID-19 virus was predominantly in locations like Asia, where strict precautionary measures were enforced, before April 2020. However, as the virus progressed, interventions in Europe and the United States became stricter by approximately September 2020.
2. As COVID-19 continues to mutate, its transmission characteristics change (scenario 2) to relatively high reproduction numbers, a short incubation period, a short infection period, and a low mortality rate. In terms of characteristics, although the rate and extent of transmission have increased, the short infection period and low mortality rate make the strictest control no longer optimal for high-cost economies, especially as vaccines become more widely available, and relaxation of restrictions will be optimal. For low-cost economies, however, tight control still maintains a positive net benefit, and thus there is an incentive to choose the tightest intervention at a later stage. This explains why, in practice, high-income countries or regions such as the United States and Europe have generally adopted a policy of gradual relaxation of restrictions in the late stages of the 2021 pandemic [58, 59]. Middle-income countries such as China, on the other hand, still chose to implement a strict control policy [58]. Hale T., et al. [58] show that China implemented a “zero COVID” approach in 2022. This policy model involves a dynamic and rigorous approach to prevention and control at each wave of an outbreak, requiring extensive nucleic acid testing, sentinel blockades, and epidemiological investigations. The policy target was “dynamic clearance instead of zero infection, aiming to maximise early detection, early treatment, and early disposal and resolutely prevent the continuous spread of the epidemic in communities”.
3. We find that mortality plays an important role in the policy choices of high-cost economies. Based on the comparison of the results of Scenario 1 with transmission characteristics of initial virus and Scenario 2 with transmission characteristics of variants in M and infection fatality rate section, high-cost economies tend to choose the most stringent interventions if the infection fatality rate is very high, both in the early stages of the outbreak and in the late stages of the virus’ mutation. In practice, in the late stages of the COVID-19 outbreak, when the infection fatality rate of the virus is much lower, the high-cost economies (generally high-income economies) are to opt for a full liberalisation of prevention and control policies. For them, at low infection fatality rate, with increased treatment technologies and vaccination coverage, the costs (including isolation direct costs, isolation indirect costs, etc.) from prevention and control will exceed the total benefits, and thus full liberalisation will be optimal. And according to our data, middle-income economies usually face relatively lower costs of prevention and control than high-income economies, such as fewer lost wages, lower isolation costs, etc., and they are able to maintain strict control policies for a longer period of time.
4. On the basis of the basic model, we further explore the extra cost of control policy. We find that as the pandemic evolves, the net benefits of high-cost economies approach zero, while low-cost economies do not. For low-cost economies, as shown in Fig. 10, in scenario 2, the net benefit is mostly around 6,000 yuan per capita when the extra economic loss from the lockdown is not considered. If the extra economic loss is more than 6,000 yuan, then the ease of the lockdown would be the optimal policy. According to existing research, a one-month lockdown policy on Shanghai in 2022 alone could lead to a 10 per cent average reduction in national real income [56], about 3,000 RMB.^6^ If multiple lockdowns are imposed on multiple cities, the losses will be even greater. This can also be evidenced from other studies. Some studies have concluded that the impact of stringent restrictions on GDP is much larger than the number of COVID deaths [60]. Moreover, COVID-19 deaths have a greater impact on GDP in advanced economies than in emerging markets and developing economies [60]. Indeed, middle-income economies such as China, which in the later stages of the pandemic outbreak were less resilient than they had been during the first round of infections, although they were able to implement a lockdown policy at lower cost than high-income economies [56].

In addition, the previous discussion is focused on mean costs and it does not take into account variations in the economic structure of countries. For instance, if a nation’s economy is systematically compatible with the lockdown policy, it incurs fewer economic damages and encounters lower supplementary expenditures during the embargo policy. Then, it is probable that a rigorous lockdown policy could be sustained for an extended duration. If a country’s economic structure is better suited for population movements or foreign trade, it is more likely to be able to lift the lockdown policy. This is because the country would face higher economic losses and additional costs under an embargo policy. Naturally, this structural adaptation changes over time as the duration of the lockdown policy expands. Additionally, various economic structures can support it for different durations, leading to an unsynchronized relaxation timing. Some studies have found that industries whose economic production and activities are less dependent on population mobility are more resilient under strict mobility controls [55]. Rationalisation of industrial structure has an uplifting effect on urban resilience [61]. Organisational resilience also affects the economy’s ability to recover from a COVID-19 pandemic shock [62]. Therefore, to reduce the economic impact of the pandemic and control policies, countries around the world need to continuously increase their economic resilience, social resilience, environmental resilience, infrastructure resilience and institutional resilience.

The limitations of this paper mainly relate to two aspects. The first is that in the calculation of costs, we did not specifically quantify the additional control costs suffered by susceptible groups besides the infected population, such as economic losses brought about by economic stagnation. This point involves many issues of macroeconomics that require further in-depth discussion. Moreover, the main purpose of this paper is to propose a model for optimal policy selection in public health emergencies based on cost-benefit analysis, simulating the impact of the main variable changes on the optimal policy choice. The second is that this paper only uses the per capita weighted average cost-benefit for model simulation without considering the population scale and structure of the economy. The main reason is that, in this model, population size does not affect the results. Additionally, to compare the cost-benefits of countries with different cost levels, consistency must be maintained in units for a more intuitive comparison. When subsequently analysing the real economic loss of the non-infected population from a macroeconomic perspective, the population scale needs to be further considered, requiring us to optimise the model in more detail.

## Conclusion

In this study, we constructed a cost-benefit model of COVID-19 prevention and control policies based on epidemic transmission and used numerical simulations to analyse the differences in policy choices among countries with different income levels. Firstly, the study discovered that both high-cost and low-cost countries mostly opted for strict lockdown measures in response to the characteristics of early-stage viruses. Viewing early viruses from a virus transmission standpoint, higher mortality rates and longer transmission cycles occurred, making quarantine policies a way to avoid the economic losses associated with large numbers of deaths in the population. Secondly, considering the features of virus transmission subsequent to mutations − faster virus transmission and lower mortality rates − high-cost countries usually ease restrictions, whilst low-cost countries tend to impose stricter embargoes. Thirdly, considering the extra economic costs of the restriction policy, we find that relaxation is also the optimal policy for low-cost countries in the late stages of the pandemic.

The conclusions have significant implications for policymakers. When making policy decisions concerning the prevention and control of public health emergencies in a country, the government should choose policies dynamically, taking full account of factors such as characteristics of virus transmission, economic structure of a country, life and health benefits of policies, and economic costs of policies. In the age of big data and the thriving development of digital technology, public health policymakers can build a cost-benefit big data model based on their own country’s data. Such a model will facilitate the real-time prediction of the health and economic benefits that prevention and control policies may yield in the short and long term.

In terms of costs, the costs of prevention and control policies play a key role in policy decision-making. Policy costs include not only direct costs, but also indirect extra economic costs. This, in turn, determines the feasibility of implementing lockdown policies, particularly in countries with unstable economic structure and industrial system heavily reliant on population mobility. The policy’s maintainability will be severely impacted, henceforth future public health policymaking must consider both the resilience of the public health system and that of the country and region’s economy and environment. It is critical for researchers to conduct a detailed investigation into the economic structure that can exhibit more resilience in the face of epidemic shocks. This is imperative for effectively responding to new public health challenges in the future.

## Data Availability

The datasets used and/or analysed during the current study are available from the corresponding author on reasonable request.

## Footnotes

1 The simplest model of epidemic, consists of three compartments: susceptible(S), infected(I), recovered(R).

2 The reason for using Disability-Adjusted Life Years (DALYs) is that it includes both years of life lost due to premature death and years lived with disability. COVID-19 not only results in death and the loss of all human capital, but also causes partial loss of human capital due to illness and its aftermath.

3 Here, to simplify the analysis, we equate the value of each individual's life and do not consider the impact of health inequality.

4 The calculation formula for YLDs refers to the algorithm provided by WHO: “YLDs were developed from a simple disease model indicating likely sequelae. YLD = I*DW*L, where I is the number of incident cases per year, DW is the disability weight and L the average duration of the disease until remission or death in years. This incidence based approach was the method undertaken in the present study.” https://www.who.int/data/gho/indicator-metadata-registry/imr-details/4647#:~:text=YLDs%20were%20developed%20from%20a%20simple%20disease%20model,the%20disease%20until%20remission%20or%20death%20in%20years.

5 In the report of Baggett TP, et al. (2020), the daily cost of alternative care sites is USD$304, the daily cost of temporary housing is USD$141. In our SEQIRD model, it simplifies the assumptions about the quarantine population and does not distinguish between infected and non-infected populations for the quarantine population. The purpose of setting up an isolated population is to calculate how many susceptible people will briefly drop out of the virus transmission pathway due to isolation. Therefore, isolated populations need not only temporary housing, but also alternative care sites. We set the direct non-medical cost by assuming that all isolated populations require varying degrees of care space needs, resulting in a cost of $304.

6 According to China's National Bureau of Statistics, China's per capita disposable income in 2022 will be RMB 36,883. Available at: http://www.stats.gov.cn/sj/zxfb/202302/t20230203_1901715.html
